# Crystal structure, Hirshfeld surface analysis and computational study of the 1:2 co-crystal formed between *N*,*N*′-bis­(pyridin-4-ylmeth­yl)ethane­diamide and 4-chloro­benzoic acid

**DOI:** 10.1107/S2056989020000572

**Published:** 2020-01-21

**Authors:** Sang Loon Tan, Edward R. T. Tiekink

**Affiliations:** a Research Centre for Crystalline Materials, School of Science and Technology, Sunway University, 47500 Bandar Sunway, Selangor Darul Ehsan, Malaysia

**Keywords:** crystal structure, oxalamide, hydrogen bonding, Hirshfeld surface analysis, computational chemistry

## Abstract

The asymmetric unit of the title 1:2 co-crystal, C_14_H_14_N_4_O_2_·2C_7_H_5_ClO_2_, comprises two half mol­ecules of oxalamide (^4^ *L*H_2_), as each is disposed about a centre of inversion, and two mol­ecules of 4-chloro­benzoic acid (CBA), each in general positions. Each ^4^ *L*H_2_ mol­ecule has a (+)anti­periplanar conformation with the pyridin-4-yl residues lying to either side of the central, planar C_2_N_2_O_2_ chromophore with the dihedral angles between the respective central core and the pyridyl rings being 68.65 (3) and 86.25 (3)°, respectively, representing the major difference between the independent ^4^ *L*H_2_ mol­ecules. The *anti* conformation of the carbonyl groups enables the formation of intra­molecular amide-N—H⋯O(amide) hydrogen bonds, each completing an *S*(5) loop. The two independent CBA mol­ecules are similar and exhibit C_6_/CO_2_ dihedral angles of 8.06 (10) and 17.24 (8)°, indicating twisted conformations. In the crystal, two independent, three-mol­ecule aggregates are formed *via* carb­oxy­lic acid-O—H⋯N(pyrid­yl) hydrogen bonding. These are connected into a supra­molecular tape propagating parallel to [100] through amide-N—H⋯O(amide) hydrogen bonding between the independent aggregates and ten-membered {⋯HNC_2_O}_2_ synthons. The tapes assemble into a three-dimensional architecture through pyridyl- and methyl­ene-C—H⋯O(carbon­yl) and CBA-C—H⋯O(amide) inter­actions. As revealed by a more detailed analysis of the mol­ecular packing by calculating the Hirshfeld surfaces and computational chemistry, are the presence of attractive and dispersive Cl⋯C=O inter­actions which provide inter­action energies approximately one-quarter of those provided by the amide-N—H⋯O(amide) hydrogen bonding sustaining the supra­molecular tape.

## Abstract 2

In the title 1:2 co-crystal, ^4^ *L*H_2_:2CBA, two independent three-mol­ecule aggregates, *i.e*. ^4^ *L*H_2_(CBA)_2_, are formed, each located about a centre of inversion and sustained by carb­oxy­lic acid-O—H⋯N(pyrid­yl) hydrogen bonding. The three-mol­ecule aggregates are connected into a supra­molecular tape by amide-N—H⋯O(amide) hydrogen bonding.

## Chemical context   

This paper describes the X-ray crystal structure determination of, and an analysis of the supra­molecular association in the 1:2 co-crystal formed between bis­(pyridin-4*-*ylmeth­yl)ethanedi­amide and 4-chloro­benzoic acid, (I). The isomeric bis­(pyridin-*n-*ylmeth­yl)ethanedi­amide mol­ecules, *i.e*. mol­ecules of the general formula *n*-NC_5_H_4_CH_2_N(H)C(=O)C(=O)CH_2_C_5_H_4_N-*n*, for *n* = 2, 3 and 4, hereafter abbreviated as *^n^L*H_2_, are of inter­est as co-crystal co-formers owing to the presence of amide and pyridyl hydrogen bonding possibilities in their mol­ecular structures (Tiekink, 2017 ▸). In a recent survey of co-crystals formed between ^4^ *L*H_2_ and carb­oxy­lic acids (Tan & Tiekink, 2020 ▸), the formation of carb­oxy­lic acid-O—H⋯N(pyrid­yl) hydrogen bonds in their co-crystals was reported to be universal with only one exception. The odd co-crystal was the 1:1 co-crystal formed between ^4^ *L*H_2_ and 2-[(4-hy­droxy­phen­yl)diazen­yl]benzoic acid (Arman *et al.*, 2009 ▸). Within the acid, an intra­molecular carb­oxy­lic acid-O—H⋯N(azo) hydrogen bond is instituted instead, leading to the formation of a *S*(6) loop, an observation entirely in accord with expectation (Etter, 1990 ▸). The remaining co-crystal structures of ^4^ *L*H_2_ with different carb­oxy­lic acids were stabilized by the expected carb­oxy­lic acid-O—H⋯N(pyrid­yl) hydrogen bonds, at both ends of the ^4^ *L*H_2_ mol­ecule. The formation of such O—H⋯N hydrogen bonding is consistent with literature precedent, which indicates a very high propensity for these hydrogen-bonding patterns between carb­oxy­lic acids and pyridyl entities, at least in the absence of competing supra­molecular synthons (Shattock *et al.*, 2008 ▸). In only one case of co-crystallization experiments of ^4^ *L*H_2_ with carb­oxy­lic acids was a salt formed owing to proton transfer, *i.e.* in the structure of [^4^ *L*H_4_][2,6-di­nitro­benzoate]_2_, where pyridinium-N—H⋯O(carboxyl­ate) hydrogen bonds are formed instead (Arman, Miller *et al.*, 2012 ▸). The title co-crystal, (I), was studied in continuation of on-going investigations of ^4^ *L*H_2_ co-crystals of carb­oxy­lic acid co-formers (Arman *et al.*, 2012 ▸, 2013 ▸, 2014 ▸; Syed *et al.*, 2016 ▸; Tan, Halcovitch *et al.*, 2019 ▸; Tan & Tiekink, 2019 ▸).
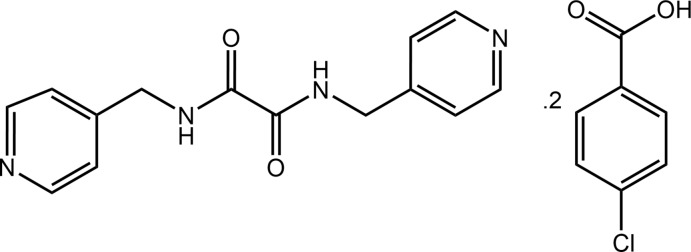


## Structural commentary   

**Figure 1:**
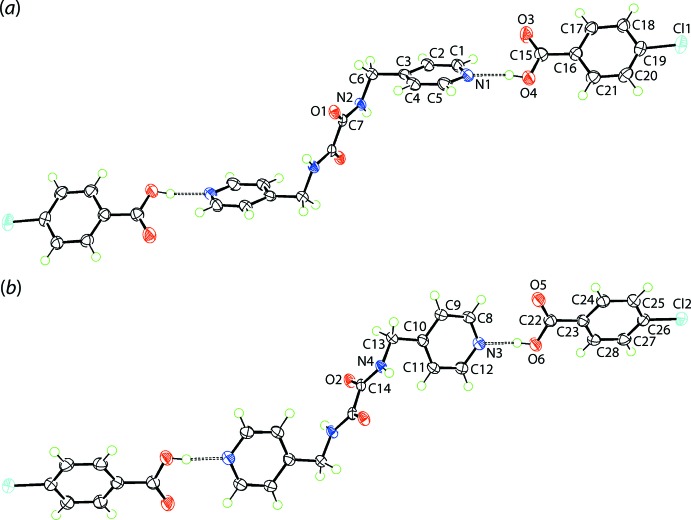
The mol­ecular structures of the two centrosymmetric three-mol­ecule aggregates in the crystal of (I) showing the atom-labelling scheme and displacement ellipsoids at the 70% probability level. In (*a*), the unlabelled atoms are related by the symmetry operation (i) 1 − *x*, 2 − *y*, − *z* and in (*b*), by (ii) 2 − *x*, 2 − *y*, − *z*.

The crystallographic asymmetric unit of (I) comprises two half mol­ecules of ^4^ *L*H_2_, each being disposed about a centre of inversion, and two mol­ecules of 4-chloro­benzoic acid (CBA), each in a general position. Pairs of ^4^ *L*H_2_ and CBA mol­ecules are connected *via* carb­oxy­lic acid-O—H⋯N(pyrid­yl) hydrogen bonding, Table 1 ▸, and with the application of symmetry, two independent, three-mol­ecule aggregates eventuate, *i.e*. ^4^ *L*H_2_(CBA)_2_, as shown in Fig. 1 ▸.

**Figure 2:**
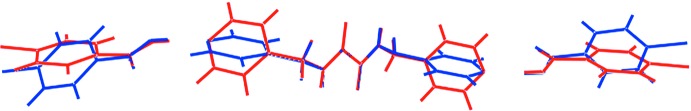
An overlay diagram of the two independent, three-mol­ecule aggregates in (I). The N1-pyrid­yl/O3-carb­oxy­lic acid (red image) and N3-pyrid­yl/O5-carb­oxy­lic acid (blue image) aggregates have been overlapped so that the central C_2_N_2_O_2_ chromophores are coincident.

As each ^4^ *L*H_2_ mol­ecule is centrosymmetric, the central C_2_N_2_O_2_ chromophore in each is strictly planar. As is usually found in these mol­ecules (Tiekink, 2017 ▸; Tan & Tiekink, 2020 ▸), the central C7—C7^i^ [1.537 (2) Å] and C14—C14^ii^ [1.539 (2) Å] bond lengths are longer than usual owing to the electronegative substituents connected to both carbon atoms [symmetry operations (i) 1 − *x*, 2 − *y*, − *z* and (ii) 2 − *x*, 2 − *y*, − *z*]. The conformation of each ^4^ *L*H_2_ mol­ecule is (+)anti­periplanar whereby the pyridin-4-yl residues lie to either side of the planar region of the mol­ecule. The dihedral angles between the respective central core and the N1- and N3-pyridyl rings are 68.65 (3) and 86.25 (3)°, respectively. This represents the greatest conformational difference between the ^4^ *L*H_2_ mol­ecules and is emphasized in the overlay diagram of Fig. 2 ▸ which shows the two independent, three-mol­ecule aggregates. Finally, the carbonyl groups are *anti*, enabling the formation of intra­molecular amide-N—H⋯O(amide) hydrogen bonds that complete *S*(5) loops, Table 1 ▸.

To a first approximation, the two independent CBA mol­ecules in (I) are similar. The dihedral angle between the benzene ring and the attached CO_2_ group is 8.06 (10)° for the O3-mol­ecule indicating a closer to co-planar mol­ecule than for the O5-mol­ecule for which the equivalent dihedral angle is 17.24 (8)°. Consistent with the carb­oxy­lic acid assignment, the C15—O3(carbon­yl) bond length of 1.2172 (17) Å is considerably shorter than the C15—O4(hy­droxy) bond of 1.3196 (16) Å; the bonds of the O5-benzoic acid follow the same trend with C22—O5 of 1.2173 (17) Å compared with C22—O6 of 1.3181 (16) Å. As seen from Fig. 2 ▸, the attached benzoic acid mol­ecules are each twisted out of the plane through the pyridyl ring they are connected to as seen in the N1-pyrid­yl/O3-carb­oxy­lic acid dihedral angle of 41.70 (4)°; the corresponding angle for the second three-mol­ecule aggregate is 35.47 (3)°.

## Supra­molecular features   

**Figure 3:**
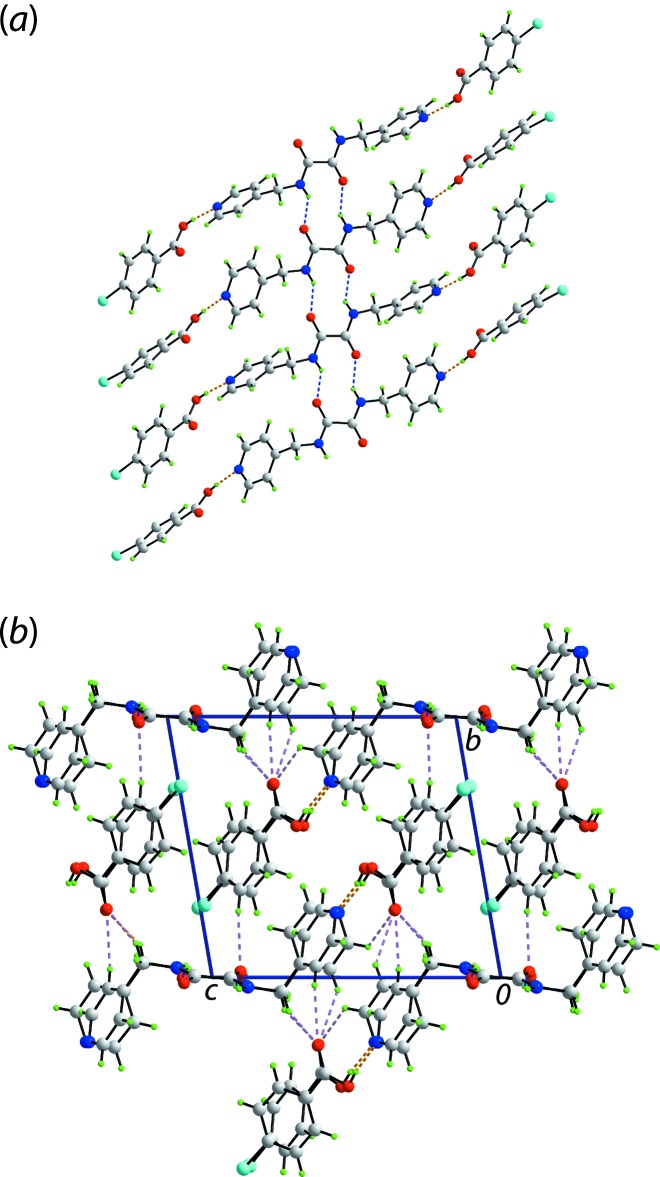
Mol­ecular packing in the crystal of (I): (*a*) supra­molecular tape comprising three-mol­ecule aggregates, each sustained by carb­oxy­lic acid-O—H⋯N(pyrid­yl) hydrogen bonding (orange dashed lines), linked by amide-N—H⋯O(amide) (blue dashed lines) hydrogen bonding and (*b*) a view of the unit-cell contents down the *a* axis with C—H⋯O inter­actions highlighted by pink dashed lines.

The formation of two independent, three-mol­ecule aggregates has already been noted above in the crystal of (I) as has the intra­molecular amide-N—H⋯O(amide) hydrogen bonds, Table 1 ▸. The carb­oxy­lic acid-O—H⋯N(pyridyl) hydrogen bond involving the O5-carb­oxy­lic acid and N3-pyridyl ring is supported by a pyridyl-NC—H⋯O(carbon­yl) contact which closes a seven-membered {⋯OCOH⋯NCH} pseudo-heterosynthon; the corresponding H⋯O separation for the O3-carb­oxy­lic acid and N1-pyridyl ring is 2.67 Å. The three-mol­ecule aggregates are connected into a supra­molecular tape along the *a* axis by amide-N—H⋯O(amide) hydrogen bonding and concatenated, centrosymmetric 10-membered {⋯HNC_2_O}_2_ synthons, Fig. 3 ▸(*a*). The tapes are consolidated into a three-dimensional architecture by pyridyl- and methyl­ene-C—H⋯O(carbon­yl) and CBA-C—H⋯O(amide) inter­actions, Fig. 3 ▸(*b*).

## Hirshfeld surface analysis   

The calculation of the Hirshfeld surfaces and two-dimensional fingerprint plots were accomplished with the program *Crystal Explorer 17* (Turner *et al.*, 2017 ▸) using procedures described in the literature (Tan, Jotani *et al.*, 2019 ▸; Jotani *et al.*, 2019 ▸). The input for the calculations were the two independent three-mol­ecule aggregates, hereafter 3M-I and 3M-II, shown in Fig. 2 ▸, whereby two chloro­benzoic acid (CBA) mol­ecules are connected to each ^4^ *L*H_2_ mol­ecule *via* carb­oxy­lic acid-O—H⋯N(pyrid­yl) hydrogen bonds. Analogous calculations were also performed on the symmetry expanded N1- and N3-oxalamide mol­ecules, hereafter ^4^ *L*H_2_-I and ^4^ *L*H_2_-II, respectively, and on the independent O3- and O5-chloro­benzoic acid mol­ecules, hereafter CBA-I and CBA-II, respectively. The *d*_norm_ distances for short contacts identified through the Hirshfeld surface analysis are given in Table 2 ▸.

**Figure 4:**
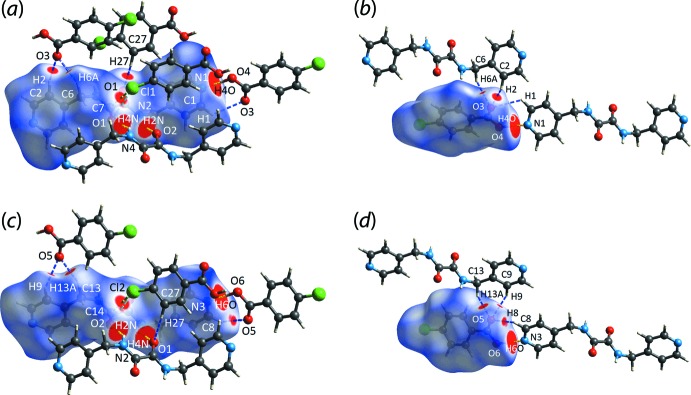
The *d*_norm_ maps showing N—H⋯O (yellow dashed lines), C—H⋯O (blue), Cl⋯C (green) and Cl⋯O (light-blue) close contacts as indicated by the corresponding red spots with varying intensities within the range of −0.0503 to 1.1157 arbitrary units for (*a*) ^4^ *L*H_2_-I, (*b*) CBA-I, (*c*) ^4^ *L*H_2_-II and (*d*) CBA-II.

Several *d*_norm_ maps showing red spots ranging from moderate to strong intensity are illustrated in Fig. 4 ▸. In particular, intense red spots indicative of strong inter­actions (Spackman & Jayatilaka, 2009 ▸) are observed for carb­oxy­lic-O4—H4*O*⋯N1(pyrid­yl) in 3M-I, carb­oxy­lic-O6–H6*O*⋯N3(pyrid­yl) in 3M-II as well as the inter­actions between amide-N2–H2*N*⋯O2(amide) and amide-N4—H4*N*⋯O1(amide) in 3M-I and 3M-II, respectively, while relatively weaker inter­actions with moderately to weakly intense red spots between amide-C7⋯Cl1, pyridyl-C1—H1⋯O3(carboxy­lic acid), pyridyl-C2—H2⋯O3(carb­oxy­lic acid), methyl­ene-C—H6*A*⋯O3(carb­oxy­lic acid), amide-O1⋯Cl1 in 3M-I, and Cl2⋯C14(amide), methyl­ene-C13—H13*A*⋯O5(carboxylic acid), pyridyl-C8—H8⋯O5(carb­oxy­lic acid), pyridyl-C9—H9⋯O5(carb­oxy­lic acid), Cl2⋯O2(amide) in 3M-II are observed. As well, spots due to benzene-C27—H27⋯O1(amide) are seen, *i.e*. providing connections between 3M-I and 3M-II.

Qualitatively, the *d*_norm_ maps for 3M-I and 3M-II exhibit similarity for the corresponding ^4^ *L*H_2_ and CBA mol­ecules with the exception of CBA-II. Pairs of CBA-II are aligned around an inversion centre with Cl2 and H25 being directly opposite each other, ostensibly forming an eight-membered heterosynthon despite the distance being longer than the cut-off value of 2.84 Å (Spek, 2020 ▸); such an alignment is not observed for CBA-I. In addition, there are other close contacts: C1—H1⋯O3, C6—H6*A*⋯O3, C7⋯Cl1, Cl2⋯C14, O1⋯Cl1 and Cl2⋯O2, which were not identified in the *PLATON* (Spek, 2020 ▸) analysis.

**Figure 5:**
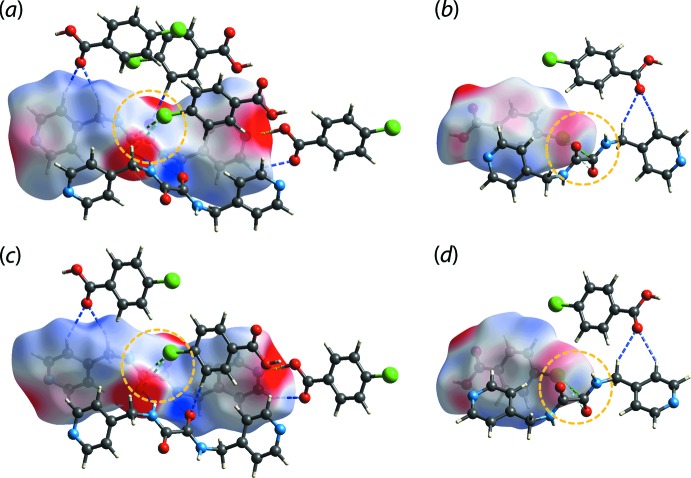
The electrostatic potential mapped onto the Hirshfeld surfaces within the isosurface value of −0.0416 to 0.0981 atomic units for (*a*) ^4^ *L*H_2_-I, (*b*) CBA-I, (*c*) ^4^ *L*H_2_-II and (*d*) CBA-II. The circles highlight the inter­actions between the electronegative sites of the amide and the chlorine atoms through the electropositive σ-hole region.

To establish the nature of the inter­molecular inter­actions, particularly for the weaker contacts, a mapping of the electrostatic potential (ESP) was performed over the Hirshfeld surfaces through DFT-B3LYP/6-31G(*d*,*p*) for the independent ^4^ *L*H_2_ and CBA mol­ecules in (I), Fig. 5 ▸. The results indicate the C1—H1⋯O3, C6—H6*A*⋯O3, C7⋯Cl1, Cl2⋯C14, O1⋯Cl1 and Cl2⋯O2 contacts are indeed electrostatic in nature, as shown from the red (electronegative) and blue (electropositive) regions on the ESP maps despite being relatively less intense when compared to those arising from the classical hydrogen bonds.

**Figure 6:**
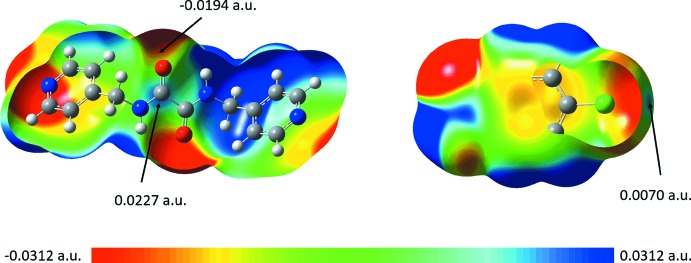
The electrostatic potential surface mapping for ^4^ *L*H_2_ and CBA as obtained from *Gaussian 16*, showing the average ESP charge on the surface of the point of contact for the Cl1/Cl2, C7/C14 and O1/O2 inter­actions. The electrostatic potential was mapped onto the isodensity surface (0.0004 a.u.) within the scale of −0.0312 to 0.0312 a.u.

ESP calculations were also performed on the individual mol­ecules through *Gaussian 16* (Frisch *et al.*, 2016 ▸) using the long-range corrected wB97XD density functional with Grimme’s D2 dispersion density functional theoretical model (Chai & Head-Gordon, 2008 ▸) coupled with Pople’s 6-311+G(*d,p*) basis set (Petersson *et al.*, 1988 ▸) in order to validate the above results. The calculations show that the individual ^4^ *L*H_2_ and CBA mol­ecules possess similar electrostatic surface potentials with the red and blue regions representing the extremities of the electrostatic potential spectrum, Fig. 6 ▸.

**Figure 7:**
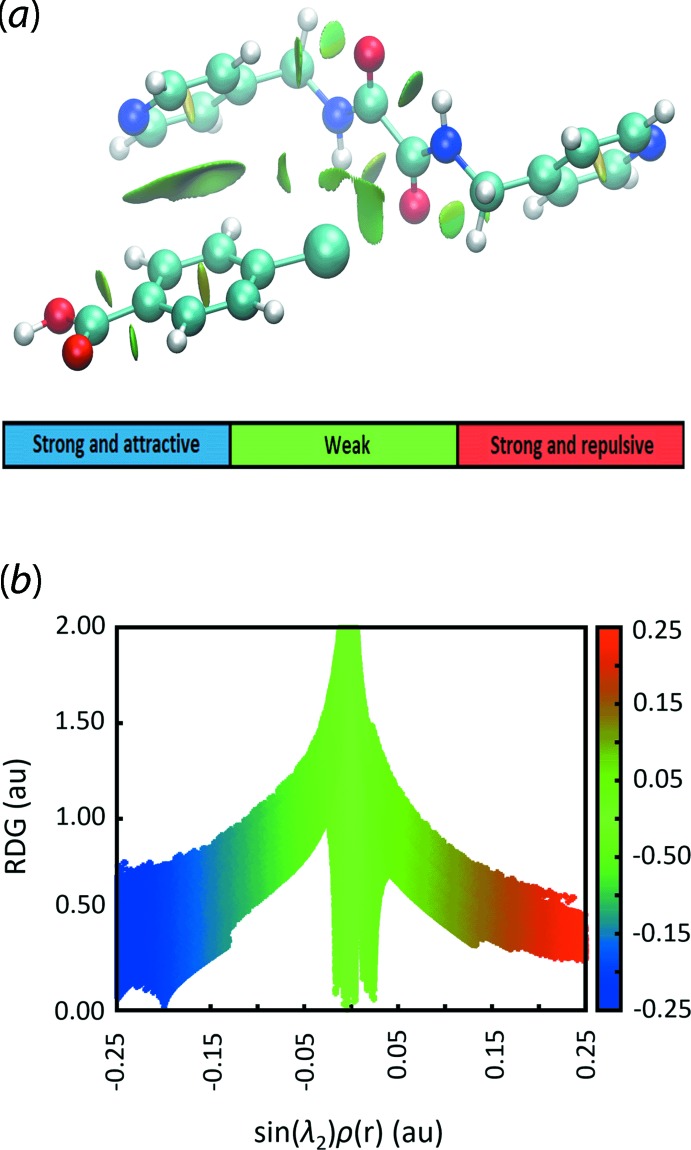
(*a*) The NCI plot highlighting the O⋯Cl and C⋯Cl contacts between ^4^ *L*H_2_-I and CBA-I mol­ecules, showing the weak, but attractive inter­actions through the green domain and (*b*) the two-dimensional reduced density gradient versus the electron density times the sign of the second Hessian eigenvalue which reveals the overall contact profile of the pairwise mol­ecules. The gradient cut-off is set at 0.4 and the colour scale is −0.25 < ρ < 0.25 a.u.

Of particular inter­est is the observation that the chlorine atom inter­acts with the amide-C=O residue through an electron-deficient σ-hole region. To complement the ESP findings on these O⋯Cl and C⋯Cl contacts, non-covalent inter­action plots were generated for the relevant pairwise mol­ecules using *NCIPLOT* (Johnson *et al.*, 2010 ▸). The results, as shown from the green domain on the isosurface between the ^4^ *L*H_2_ and CBA mol­ecules in Fig. 7 ▸, indicate that those inter­actions are weakly attractive (Contreras-García *et al.*, 2011 ▸). The calculated electrostatic potential charge on the surface at the point of contacts calculated with *Crystal Explorer 17* employing B3LYP/6-31G(*d*,*p*) are comparable to the data obtained from *Gaussian 16*, in which Cl1, O1, Cl2 and O2 possess charges of +0.0054, −0.0147, +0.0054 and −0.0125 atomic units (a.u.), respectively; while the C7 and C14 atoms each exhibit a weak electrostatic potential charge of +0.0251 and +0.0263 a.u., respectively. Therefore, the C7⋯Cl1 and C14⋯Cl2 inter­actions are dispersive in nature. On the other hand, the apparent charge complementarity between the Cl2 and H25 atoms, which align around a centre of inversion as described above, indicate the existence of an electrostatic inter­action between two CBA-II mol­ecules, Fig. 5 ▸(*d*).

**Figure 8:**
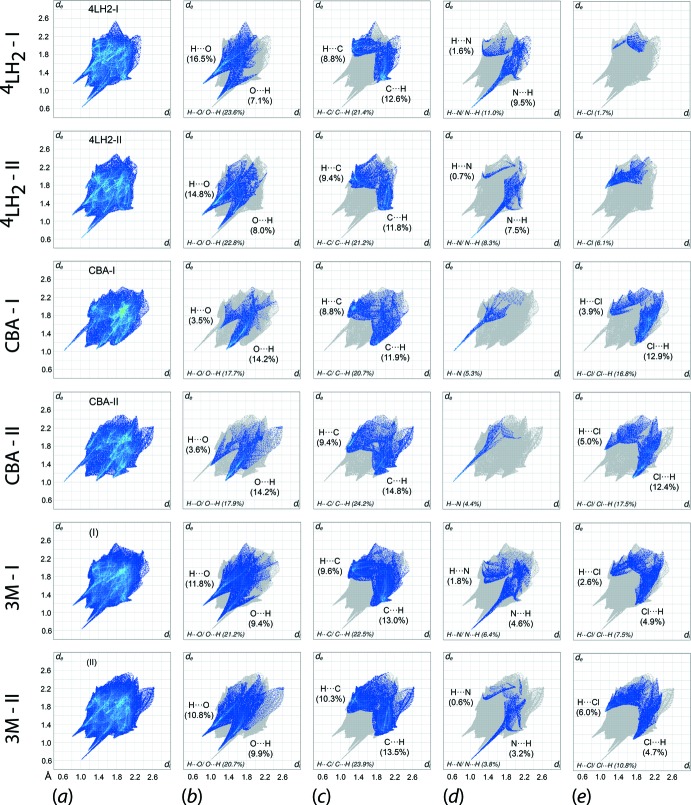
(*a*) The overall two-dimensional fingerprint plots for ^4^ *L*H_2_-I, ^4^ *L*H_2_-II, CBA-I, CBA-II, 3M-I and 3M-II, and those delineated into (*b*) H⋯O/O⋯H, (*c*) H⋯N/N⋯H, (*d*) H⋯C/C⋯H and (*e*) H⋯Cl/Cl⋯H contacts, with the percentage contributions specified within each plot.

The two-dimensional fingerprint plots were generated in order to qu­antify the close contacts for ^4^ *L*H_2_-I, ^4^ *L*H_2_-II, CBA-I, CBA-II, 3M-I and 3M-II. The overall fingerprint plots for the specified mol­ecules/aggregates are shown in Fig. 8 ▸(*a*) and those decomposed into H⋯O/O⋯H/ H⋯C/C⋯H, H⋯N/N⋯H and H⋯Cl/Cl⋯H plots are shown in Fig. 8 ▸(*b*)-(*e*).

The overall fingerprint plot of the individual components and the corresponding three-mol­ecule aggregates exhibit a paw-like profile with asymmetric spikes indicating the inter-dependency of the inter­molecular inter­actions between mol­ecules to sustain the packing. The 3M-I and 3M-II aggregates display almost identical fingerprint profiles which, upon decomposition, can be delineated into H⋯H [32.5% for 3M-I and 30.1% for 3M-II; not illustrated], H⋯C/C⋯H [22.5 and 23.9%, respectively], H⋯O/O⋯H [21.2 and 20.7%], H⋯Cl/Cl⋯H [7.5 and 10.8%], H⋯N/N⋯H [6.4 and 3.8%] and other minor contacts [10.0 and 10.7%]. A detailed analysis on the corresponding decomposed fingerprint plots shows that only the H⋯O/O⋯H and H⋯N/N⋯H contacts for both 3M-I and 3M-II as well as H⋯Cl/Cl⋯H for 3M-II have *d*_i_ + *d*_e_ distances shorter than the sum of the respective van der Waals radii of 2.61, 2.64 and 2.84 Å (adjusted to neutron values). For 3M-I, the *d*_i_ + *d*_e_ values for the H⋯O/O⋯H and H⋯N contacts are, respectively, tipped at ∼1.98, ∼1.95 and ∼1.68 Å, and are attributed to (inter­nal)-N2—H2*N*⋯O2-(external), (inter­nal)-O1⋯H4*N*-(external) and (inter­nal)-N1⋯H4*O*-(external) contacts, respectively. The analogous contacts for 3M-II are tipped at 1.95 Å for (inter­nal)-H4*N*⋯O1-(external), ∼1.98 Å for (inter­nal)-O2⋯H2*N*-(external) and ∼1.64 Å for (inter­nal)-N3⋯H6*O*-(external). For H⋯Cl/Cl⋯H in 3M-II, the contacts are each tipped at ∼2.80 Å owing to the pair of (inter­nal)-H25⋯Cl2-(external) and (inter­nal)-Cl2⋯H25-(external) inter­actions. As for the H⋯H and H⋯C/ C⋯H contacts, their *d*_i_ + *d*_e_ distances are longer than the sum of their respective van der Waals radii of 2.18 and 2.79 Å, and hence contribute little to the overall packing of the crystal despite providing the predominant surface contacts.

The individual ^4^ *L*H_2_-I and ^4^ *L*H_2_-II mol­ecules exhibit similar fingerprint profiles with only slight differences in the contact distributions. In order of dominance, these are H⋯H (36.3% for ^4^ *L*H_2_-I and 33.8% for ^4^ *L*H_2_-II), H⋯O/O⋯H (23.6 and 22.8%, respectively), H⋯C/C⋯H (21.4 and 21.2%), H⋯N/N⋯H (11.0 and 8.3%), H⋯Cl (1.7 and 6.1%) and other minor contacts (6.0 and 7.8%). There is no major deviations in the *d*_i_ + *d*_e_ distances *cf*. 3M-1 and 3M-II, with only the H⋯O/O⋯H as well as N⋯H contacts being shorter than the sums of their respective van der Waals radii. Each of ^4^ *L*H_2_-I and ^4^ *L*H_2_-II have *d*_i_ + *d*_e_ of about 1.98 Å for H⋯O/O⋯H and ∼1.64 Å for N⋯H contacts.

As for the individual CBA-I and CBA-II mol­ecules, major contacts comprise H⋯H (23.7% for CBA-I and 22.1% for CBA-II), H⋯C/C⋯H (20.7 and 24.2%, respectively), H⋯O/O⋯H (17.7 and 17.9%), H⋯Cl/Cl⋯H (16.8 and 17.5%), H⋯N (5.3 and 4.4%) and other minor contacts (15.7 and 13.9%). A detailed analysis of the corresponding contacts shows all major inter­actions for CBA-I and CBA-II are more inclined toward (inter­nal)-*X*⋯H-(external) rather than (inter­nal)-H⋯*X*-(external), as evidenced most notably from the distribution for O⋯H (CBA-I: 14.2%; CBA-II: 14.2%) *versus* H⋯O (CBA-I: 3.5%; CBA-II: 3.6%) and Cl⋯H (CBA-I: 12.9%; CBA-II: 12.4%) *vs* H⋯Cl (CBA-I: 3.9%; CBA-II: 5.0%). The inclination arises due to the lack of hydrogen-bond donor atoms in the CBA-I and CBA-II mol­ecules, other than the carb­oxy­lic acid groups, so they act primarily as hydrogen-bond acceptors. Among the contacts, O⋯H and H⋯N for CBA-I have *d*_i_ + *d*_e_ distances of ∼2.40 and ∼1.64 Å, respectively, each being shorter than the sum of the respective van der Waals radii, while the same is true for H⋯O/O⋯H, H⋯N and H⋯Cl/Cl⋯H contact for CBA-II with *d*_i_ + *d*_e_ distances of ∼2.38, ∼1.62 and ∼2.82 Å, respectively.

## Computational chemistry   

The calculation of the inter­action energies for all pairwise inter­acting mol­ecules was performed through *Crystal Explorer 17* (Turner *et al.*, 2017 ▸) based on the method reported previously (Tan, Jotani *et al.*, 2019 ▸) in order to study the strength of each inter­action identified from the Hirshfeld surface analysis. The calculations showed that the ten-membered synthons formed between ^4^ *L*H_2_-I and ^4^ *L*H_2_-II through amide-N2—H2*N*⋯O2(amide) and amide-N4—H4*N*⋯O1(amide) hydrogen bonds has the greatest energy among all close contacts present in the crystal with an inter­action energy (*E*_int_) of −61.9 kJ mol^−1^. This is followed by the seven-membered heterosynthon formed between ^4^ *L*H_2_-II and CBA-II through the carb­oxy­lic acid-O4—H4*O*⋯N1(pyrid­yl) hydrogen bond with the supporting pyridyl-C—H8⋯O5(carbon­yl) contact so that *E*_int_ = −52.0 kJ mol^−1^. For the analogous contact between ^4^ *L*H_2_-I and CBA-I but lacking the supporting pyridyl-C—H⋯O5(carbon­yl) contact, it is gratifying to note the inter­action energy is correspondingly less, *i.e. E*_int_ = −49.4 kJ mol^−1^. The inter­actions between amide-C7⋯Cl1 and amide-O1⋯Cl1, summing to *E*_int_ of −16.6 kJ mol^−1^, are also significant, as are the inter­actions between methyl­ene-C–H6*A*⋯O3(amide) and pyridyl-C2–H2⋯O3(amide) with *E*_int_ = −15.8 kJ mol^−1^. The equivalent inter­actions surrounding the ^4^ *L*H_2_-II mol­ecule follow the same trends and give similar energies, Table 3 ▸. The benzoic-C25—H25⋯Cl2 dimer arising from the connection between two CBA-II mol­ecules is weakly inter­acting with *E*_int_ of −8.7 kJ mol^−1^. Finally, the C27—H27⋯O1(amide) inter­action exhibits an *E*_int_ of −20.4 kJ mol^−1^.

**Figure 9:**
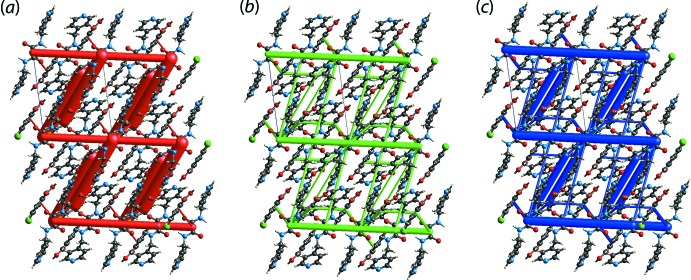
Perspective views of the energy frameworks of (I), showing the (*a*) electrostatic force, (*b*) dispersion force and (*c*) total energy. The radius of the cylinders is proportional to the relative strength of the corresponding energies and they were adjusted to the same scale factor of 100 with a cut-off value of 8 kJ mol^−1^ within a 2 × 2 × 2 unit cells.

The crystal of (I) is mainly governed by electrostatic forces (*E*_ele_) as highlighted by the rod-shaped energy framework with a zigzag topology due to the combination of several strong inter­actions, Fig. 9 ▸(*a*). Specifically, the combination of inter­actions between ^4^ *L*H_2_-I and CBA-I through the terminal O4—H4*O*⋯N1 hydrogen bonding as well as between ^4^ *L*H_2_-II and CBA-II *via* O6—H6*O*⋯N3 and C8—H8⋯O5 inter­actions leads to the formation of the core framework parallel to (101). The overall *E*_ele_ of these inter­actions is much greater than that associated with the ten-membered synthons formed by a combination of N2—H2*N*⋯O2 and N4—H4*N*⋯O1 hydrogen bonds as evidenced from the relatively small rod radius in the energy model of the latter inter­actions, which align in a parallel fashion along the *b* axis, Fig. 9 ▸(*a*).

Apart from the electrostatic forces, the crystal is also sustained by substantial dispersion forces, which are mainly associated with the ten-membered {⋯HNC_2_O}_2_ synthon along with the peripheral C7⋯Cl1/O1⋯Cl1 and C14⋯Cl2/O2⋯Cl2 inter­actions which lead to a ladder-like topology, Fig. 9 ▸(*b*). The combination of the electrostatic and dispersion forces results in an enhancement of the influence of the ten-membered synthons which supersedes the energy force for the terminal carb­oxy­lic acid-O—H⋯N(pyrid­yl) hydrogen bonds as seen in the total energy framework, Fig. 9 ▸(*c*).

## Database survey   

The formation of carb­oxy­lic acid-O—H⋯N(pyrid­yl) hydrogen bonds, involving both pyridyl rings, leading to three-mol­ecule aggregates, is an almost universal trait when co-crystals are formed between ^4^ *L*H_2_ and mono-functional carb­oxy­lic acids; one exception was noted in the *Chemical context*. A different situation pertains when bi-functional carb­oxy­lic acids are employed in co-crystal formation. In these circumstances, *e.g*. when the carb­oxy­lic acid is bis­(carb­oxy­meth­yl)urea and diglycineoxamide (Nguyen *et al.*, 2001 ▸), two-dimensional sheets result, owing to strands of {⋯HO_2_C-*R*-CO_2_H⋯^4^ *L*H_2_⋯HO_2_C-*R*-CO_2_H⋯}_*n*_ being connected by almost orthogonal tapes comprising ten-membered {⋯HNC_2_O}_2_ synthons provided by the ^4^ *L*H_2_ mol­ecules. These are reinforced by hydrogen bonding afforded by the *R* residues of the bi-functional carb­oxy­lic acids, *e.g*. linked by six-membered synthons {⋯HNCNH⋯O} provided by the urea bridges in the case of bis­(carb­oxy­meth­yl)urea (Nguyen *et al.*, 2001 ▸). Clearly, scope remains for the development of novel supra­molecular architectures in co-crystals comprising ^4^ *L*H_2_ and multi-functional carb­oxy­lic acids.

## Synthesis and crystallization   

The precursor, *N*,*N*′-bis­(pyridin-4-ylmeth­yl)oxalamide (^4^ *L*H_2_) was prepared according to a literature procedure: m.p.: 486.3–487.6 K; lit. 486–487 K (Nguyen *et al.*, 1998 ▸). 4-Chloro­benzoic acid (Merck) was reagent grade and used as received without further purification. The co-former ^4^ *L*H_2_ (0.271 g, 0.001 mol) was mixed with 4-chloro­benzoic acid (0.157 g, 0.001 mol) and the mixture was then ground for 15 min in the presence of a few drops of methanol. The procedure was repeated twice. Colourless blocks were obtained through careful layering of toluene (1 ml) on an *N*,*N*-di­methyl­formamide (1 ml) solution of the ground mixture. M.p.: 456.9–458.6 K. IR (cm^−1^): 3211 ν(N—H), 3052—2935 ν(C—H), 1669–1604 ν(C=O), 1492 ν(C=C), 1419 ν(C—N), 794 ν(C—Cl).

## Refinement   

Crystal data, data collection and structure refinement details are summarized in Table 4 ▸. The carbon-bound H atoms were placed in calculated positions (C—H = 0.95–0.99 Å) and were included in the refinement in the riding model approximation, with *U*_iso_(H) set to 1.2*U*_eq_(C). The oxygen- and nitro­gen-bound H atoms were located from a difference-Fourier map and refined with O—H = 0.84±0.01 Å and N—H = 0.88±0.01 Å, respectively, and with *U*_iso_(H) set to 1.5*U*_eq_(O) or 1.2*U*_eq_(N).

## Supplementary Material

Crystal structure: contains datablock(s) I, global. DOI: 10.1107/S2056989020000572/hb7889sup1.cif

Structure factors: contains datablock(s) I. DOI: 10.1107/S2056989020000572/hb7889Isup2.hkl

Supporting information file. DOI: 10.1107/S2056989020000572/hb7889Isup3.cml

CCDC reference: 1978104

Additional supporting information:  crystallographic information; 3D view; checkCIF report

## Figures and Tables

**Table 1:** Hydrogen-bond geometry (Å, °)

*D*—H⋯*A*	*D*—H	H⋯*A*	*D*⋯*A*	*D*—H⋯*A*
N2—H2*N*⋯O1^i^	0.88 (1)	2.35 (2)	2.7088 (14)	105 (1)
N4—H4*N*⋯O2^ii^	0.87 (1)	2.30 (2)	2.7028 (14)	108 (1)
O4—H4*O*⋯N1	0.85 (2)	1.82 (2)	2.6559 (15)	171 (2)
O6—H6*O*⋯N3	0.85 (2)	1.80 (2)	2.6419 (15)	172 (2)
C8—H8⋯O5	0.95	2.53	3.1708 (17)	125
N2—H2*N*⋯O2^iii^	0.88 (1)	2.11 (1)	2.8800 (13)	146 (1)
N4—H4*N*⋯O1	0.87 (1)	2.07 (1)	2.7959 (13)	141 (1)
C2—H2⋯O3^iv^	0.95	2.53	3.3597 (17)	146
C6—H6*A*⋯O3^iv^	0.99	2.59	3.5262 (17)	157
C9—H9⋯O5^v^	0.95	2.59	3.3701 (17)	140
C13—H13*A*⋯O5^v^	0.99	2.48	3.4006 (16)	155
C27—H27⋯O1^vi^	0.95	2.53	3.4505 (16)	163

Symmetry codes: (i) 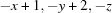; (ii) 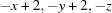; (iii) 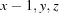; (iv) 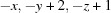; (v) 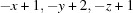; (vi) 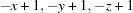.

**Table 2:** A summary of short inter­atomic contacts (Å) in (I)^*a*^

Contact	Distance	Symmetry operation
O5⋯H9	2.49	1 − *x*, 2 − *y*, 1 − *z*
O5⋯H13*A*	2.40	1 − *x*, 2 − *y*, 1 − *z*
Cl2⋯C14	3.21	−1 + *x*, −1 + *y*, 1 + *z*
Cl2⋯O2	3.22	−1 + *x*, −1 + *y*, 1 + *z*
O1⋯H27	2.92	1 − *x*, 1 − *y*, 1 − *z*
O1⋯H4*N* ^*b*^	1.96	*x*, *y*, *z*
O2⋯H2*N* ^*b*^	2.00	−1 + *x*, *y*, *z*
O5⋯H8	2.46	*x*, *y*, *z*
N3⋯H6*O* ^*b*^	1.67	*x*, *y*, *z*
O3⋯H2	2.42	1 − *x*, 2 − *y*, 1 − *z*
O3⋯H6*A*	2.51	1 − *x*, 2 − *y*, 1 − *z*
C7⋯Cl1	3.25	*x*, 1 + *y*, −1 + *z*
N1⋯H4*O* ^*b*^	1.68	−1 + *x*, *y*, *z*
O3⋯H1	2.60	−1 + *x*, *y*, *z*
O1⋯Cl1	3.24	*x*, 1 + *y*, −1 + *z*

Notes: (*a*) The inter­atomic distances are calculated in *Crystal Explorer 17* (Turner *et al.*, 2017 ▸) whereby the *X*—H bond lengths are adjusted to their neutron values; (*b*) these inter­actions correspond to conventional hydrogen bonds.

**Table 3:** A summary of inter­action energies (kJ mol^1^) calculated for (I)

Contact	*E*_ele_	*E*_pol_	*E*_dis_	*E*_rep_	*E*_tot_	Symmetry operation
N2—H2*N*⋯O2/						
N4—H4*N*⋯O1	−51.0	−12.4	−49.4	71.6	−61.9	*x*, *y*, *z*
O4—H4*O*⋯N1/						
C1—H1⋯O3	−84.3	−20.1	−12.7	106.3	−49.4	−1 + *x*, *y*, *z*
O6—H6*O*⋯N3/						
C8—H8⋯O5	−90.9	−21.4	−13.1	115.6	−52.0	*x*, *y*, *z*
C7⋯Cl1/ O1⋯Cl1	−5.3	−1.0	−25.4	19.3	−16.6	*x*, 1 + *y*, −1 + *z*
C6—H6*A*⋯O3/						
C2—H2⋯O3	−11.9	−3.2	−12.5	16.5	−15.8	1 − *x*, 2 − *y*, 1 − *z*
C9—H9⋯O5/						
C13—H13*A*⋯O5	−12.8	−3.6	−13.3	18.5	−16.3	1 − *x*, 2 − *y*, 1 − *z*
C14⋯Cl2/ O2⋯Cl2	−6.6	−0.8	−27.3	27.2	−14.5	−1 + *x*, −1 + *y*, 1 + *z*
C25—H25⋯Cl2	−6.4	−0.7	−13.5	16.7	−8.7	−*x*, − *y*, − *z*
C27—H27⋯O1	−10.4	−1.6	−23.4	19.7	−20.4	1 − *x*, 1 − *y*, 1 − *z*

**Table 4:** Experimental details

Crystal data	
Chemical formula	C_14_H_14_N_4_O_2_·2C_7_H_5_ClO_2_
*M*_r_	583.41
Crystal system, space group	Triclinic, *P* 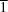
Temperature (K)	100
*a*, *b*, *c* (Å)	9.9401 (2), 11.2002 (2), 12.3308 (3)
α, β, γ (°)	78.871 (2), 78.816 (2), 81.992 (2)
*V* (Å^3^)	1313.98 (5)
*Z*	2
Radiation type	Cu *K*α
μ (mm^−1^)	2.67
Crystal size (mm)	0.13 × 0.04 × 0.03

Data collection	
Diffractometer	XtaLAB Synergy, Dualflex, AtlasS2
Absorption correction	Gaussian (*CrysAlis PRO*; Rigaku OD, 2018 ▸)
*T*_min_, *T*_max_	0.847, 1.000
No. of measured, independent and observed [*I* > 2σ(*I*)] reflections	33133, 5486, 4814
*R*_int_	0.034
(sin θ/λ)_max_ (Å^−1^)	0.631

Refinement	
*R*[*F* ^2^ > 2σ(*F* ^2^)], *wR*(*F* ^2^), *S*	0.032, 0.090, 1.03
No. of reflections	5486
No. of parameters	373
No. of restraints	4
H-atom treatment	H atoms treated by a mixture of independent and constrained refinement
Δρ_max_, Δρ_min_ (e Å^−3^)	0.29, −0.33

Computer programs: *CrysAlis PRO* (Rigaku OD, 2018 ▸), *SHELXS* (Sheldrick, 2015*a* ▸), *SHELXL2017* (Sheldrick, 2015*b* ▸), *ORTEP-3 for Windows* (Farrugia, 2012 ▸), *DIAMOND* (Brandenburg, 2006 ▸) and *publCIF* (Westrip, 2010 ▸).

## supplementary crystallographic information

### Crystal data

**Table:**

C_14_H_14_N_4_O_2_·2C_7_H_5_ClO_2_	*Z* = 2
*M**_r_* = 583.41	*F*(000) = 604
Triclinic, *P*1	*D*_x_ = 1.475 Mg m^−^^3^
*a* = 9.9401 (2) Å	Cu *K*α radiation, λ = 1.54184 Å
*b* = 11.2002 (2) Å	Cell parameters from 14644 reflections
*c* = 12.3308 (3) Å	θ = 3.7–76.1°
α = 78.871 (2)°	µ = 2.67 mm^−^^1^
β = 78.816 (2)°	*T* = 100 K
γ = 81.992 (2)°	Plate, colourless
*V* = 1313.98 (5) Å^3^	0.13 × 0.04 × 0.03 mm

### Data collection

**Table:**

XtaLAB Synergy, Dualflex, AtlasS2 diffractometer	5486 independent reflections
Radiation source: micro-focus sealed X-ray tube	4814 reflections with *I* > 2σ(*I*)
Detector resolution: 5.2558 pixels mm^-1^	*R*_int_ = 0.034
ω scans	θ_max_ = 76.5°, θ_min_ = 3.7°
Absorption correction: gaussian (CrysAlis PRO; Rigaku OD, 2018)	*h* = −9→12
*T*_min_ = 0.847, *T*_max_ = 1.000	*k* = −14→14
33133 measured reflections	*l* = −15→15

### Refinement

**Table:**

Refinement on *F*^2^	Primary atom site location: structure-invariant direct methods
Least-squares matrix: full	Secondary atom site location: difference Fourier map
*R*[*F*^2^ > 2σ(*F*^2^)] = 0.032	Hydrogen site location: mixed
*wR*(*F*^2^) = 0.090	H atoms treated by a mixture of independent and constrained refinement
*S* = 1.03	*w* = 1/[σ^2^(*F*_o_^2^) + (0.0502*P*)^2^ + 0.3813*P*] where *P* = (*F*_o_^2^ + 2*F*_c_^2^)/3
5486 reflections	(Δ/σ)_max_ = 0.002
373 parameters	Δρ_max_ = 0.29 e Å^−^^3^
4 restraints	Δρ_min_ = −0.33 e Å^−^^3^

### Special details

**Table:**

Geometry. All esds (except the esd in the dihedral angle between two l.s. planes) are estimated using the full covariance matrix. The cell esds are taken into account individually in the estimation of esds in distances, angles and torsion angles; correlations between esds in cell parameters are only used when they are defined by crystal symmetry. An approximate (isotropic) treatment of cell esds is used for estimating esds involving l.s. planes.

### Fractional atomic coordinates and isotropic or equivalent isotropic displacement parameters (Å^2^)

**Table:**

	*x*	*y*	*z*	*U*_iso_*/*U*_eq_	
O1	0.60739 (8)	0.97776 (8)	0.10123 (7)	0.01871 (18)	
N1	0.18629 (11)	0.75161 (10)	0.46595 (9)	0.0226 (2)	
N2	0.37710 (10)	1.03739 (10)	0.11754 (9)	0.0171 (2)	
H2N	0.3042 (12)	1.0441 (15)	0.0858 (13)	0.021*	
C1	0.10938 (13)	0.85755 (12)	0.43978 (11)	0.0219 (3)	
H1	0.014870	0.864969	0.473525	0.026*	
C2	0.16098 (12)	0.95678 (12)	0.36591 (11)	0.0200 (2)	
H2	0.102752	1.030329	0.349550	0.024*	
C3	0.29944 (12)	0.94745 (11)	0.31582 (10)	0.0179 (2)	
C4	0.38016 (13)	0.83804 (12)	0.34405 (11)	0.0213 (3)	
H4	0.475365	0.828721	0.312628	0.026*	
C5	0.32023 (13)	0.74264 (12)	0.41854 (11)	0.0235 (3)	
H5	0.376045	0.668080	0.436755	0.028*	
C6	0.35802 (12)	1.05429 (12)	0.23434 (11)	0.0197 (2)	
H6A	0.295105	1.129610	0.244075	0.024*	
H6B	0.447977	1.065548	0.251964	0.024*	
C7	0.50027 (11)	1.00274 (10)	0.06174 (10)	0.0146 (2)	
O2	1.10206 (8)	0.99617 (8)	0.10296 (7)	0.02007 (19)	
N3	0.69677 (11)	0.75172 (10)	0.47531 (9)	0.0211 (2)	
N4	0.86920 (10)	1.03345 (10)	0.11304 (9)	0.0173 (2)	
H4N	0.8027 (13)	1.0328 (15)	0.0771 (12)	0.021*	
C8	0.65934 (13)	0.86787 (12)	0.49045 (11)	0.0216 (3)	
H8	0.599965	0.882524	0.557915	0.026*	
C9	0.70303 (12)	0.96737 (12)	0.41257 (11)	0.0202 (2)	
H9	0.674340	1.048201	0.426901	0.024*	
C10	0.78943 (11)	0.94780 (11)	0.31302 (10)	0.0174 (2)	
C11	0.82958 (12)	0.82740 (12)	0.29757 (11)	0.0210 (3)	
H11	0.889290	0.810106	0.231079	0.025*	
C12	0.78155 (13)	0.73269 (12)	0.38030 (11)	0.0221 (3)	
H12	0.810108	0.650822	0.368968	0.027*	
C13	0.83531 (12)	1.05668 (11)	0.22714 (10)	0.0186 (2)	
H13A	0.760868	1.125137	0.231071	0.022*	
H13B	0.917196	1.082945	0.246930	0.022*	
C14	0.99697 (11)	1.00732 (11)	0.06123 (10)	0.0154 (2)	
Cl1	−0.37720 (3)	0.26656 (3)	1.00591 (3)	0.02890 (10)	
O3	−0.07627 (10)	0.73883 (9)	0.67521 (9)	0.0304 (2)	
O4	0.03544 (10)	0.58218 (9)	0.59372 (8)	0.0268 (2)	
H4O	0.0841 (18)	0.6388 (14)	0.5594 (16)	0.040*	
C15	−0.06010 (13)	0.63083 (12)	0.66763 (11)	0.0222 (3)	
C16	−0.14493 (12)	0.53923 (12)	0.74585 (11)	0.0205 (3)	
C17	−0.23844 (13)	0.57765 (12)	0.83541 (11)	0.0216 (3)	
H17	−0.252419	0.661813	0.841895	0.026*	
C18	−0.31121 (13)	0.49417 (12)	0.91512 (11)	0.0220 (3)	
H18	−0.374727	0.520178	0.976363	0.026*	
C19	−0.28935 (13)	0.37174 (12)	0.90357 (12)	0.0217 (3)	
C20	−0.19944 (13)	0.33124 (12)	0.81370 (12)	0.0240 (3)	
H20	−0.187517	0.247331	0.806467	0.029*	
C21	−0.12741 (13)	0.41610 (12)	0.73471 (12)	0.0230 (3)	
H21	−0.065666	0.390163	0.672537	0.028*	
Cl2	0.09083 (4)	0.27800 (3)	0.98266 (3)	0.03057 (10)	
O5	0.42589 (10)	0.74219 (9)	0.67193 (9)	0.0298 (2)	
O6	0.56489 (9)	0.58098 (9)	0.61812 (8)	0.0242 (2)	
H6O	0.6094 (17)	0.6374 (14)	0.5776 (14)	0.036*	
C22	0.45469 (13)	0.63220 (12)	0.67727 (11)	0.0205 (3)	
C23	0.36560 (12)	0.54182 (12)	0.75218 (11)	0.0194 (2)	
C24	0.22992 (13)	0.58221 (12)	0.79557 (11)	0.0221 (3)	
H24	0.196233	0.665934	0.777238	0.026*	
C25	0.14420 (13)	0.50108 (12)	0.86510 (11)	0.0227 (3)	
H25	0.051448	0.527996	0.893646	0.027*	
C26	0.19648 (14)	0.37974 (12)	0.89221 (11)	0.0223 (3)	
C27	0.33072 (14)	0.33723 (12)	0.85065 (12)	0.0237 (3)	
H27	0.364433	0.253713	0.870218	0.028*	
C28	0.41476 (13)	0.41920 (12)	0.77991 (11)	0.0212 (3)	
H28	0.506744	0.391414	0.750081	0.025*	

### Atomic displacement parameters (Å^2^)

**Table:**

	*U*^11^	*U*^22^	*U*^33^	*U*^12^	*U*^13^	*U*^23^
O1	0.0124 (4)	0.0244 (4)	0.0184 (4)	−0.0031 (3)	−0.0020 (3)	−0.0012 (3)
N1	0.0252 (5)	0.0229 (6)	0.0182 (5)	−0.0028 (4)	0.0006 (4)	−0.0039 (4)
N2	0.0126 (4)	0.0213 (5)	0.0165 (5)	−0.0018 (4)	−0.0001 (4)	−0.0036 (4)
C1	0.0181 (5)	0.0265 (7)	0.0199 (6)	−0.0024 (5)	0.0006 (5)	−0.0049 (5)
C2	0.0179 (6)	0.0229 (6)	0.0181 (6)	0.0003 (5)	−0.0019 (5)	−0.0041 (5)
C3	0.0184 (5)	0.0217 (6)	0.0145 (5)	−0.0025 (4)	−0.0016 (4)	−0.0063 (5)
C4	0.0189 (6)	0.0246 (6)	0.0192 (6)	0.0007 (5)	−0.0001 (5)	−0.0062 (5)
C5	0.0256 (6)	0.0223 (6)	0.0202 (6)	0.0029 (5)	−0.0018 (5)	−0.0041 (5)
C6	0.0198 (6)	0.0208 (6)	0.0179 (6)	−0.0033 (5)	0.0015 (5)	−0.0061 (5)
C7	0.0134 (5)	0.0125 (5)	0.0166 (6)	−0.0038 (4)	−0.0002 (4)	0.0002 (4)
O2	0.0128 (4)	0.0279 (5)	0.0189 (4)	−0.0032 (3)	−0.0018 (3)	−0.0028 (4)
N3	0.0195 (5)	0.0227 (5)	0.0200 (5)	−0.0023 (4)	−0.0010 (4)	−0.0031 (4)
N4	0.0118 (4)	0.0227 (5)	0.0168 (5)	−0.0019 (4)	−0.0011 (4)	−0.0036 (4)
C8	0.0219 (6)	0.0249 (6)	0.0179 (6)	−0.0028 (5)	0.0006 (5)	−0.0069 (5)
C9	0.0196 (6)	0.0207 (6)	0.0202 (6)	−0.0014 (5)	−0.0001 (5)	−0.0070 (5)
C10	0.0124 (5)	0.0220 (6)	0.0185 (6)	−0.0020 (4)	−0.0026 (4)	−0.0047 (5)
C11	0.0179 (5)	0.0227 (6)	0.0205 (6)	−0.0003 (5)	0.0024 (5)	−0.0062 (5)
C12	0.0202 (6)	0.0199 (6)	0.0247 (7)	−0.0001 (5)	0.0004 (5)	−0.0057 (5)
C13	0.0161 (5)	0.0202 (6)	0.0184 (6)	−0.0020 (4)	0.0015 (4)	−0.0053 (5)
C14	0.0134 (5)	0.0142 (5)	0.0171 (6)	−0.0036 (4)	−0.0005 (4)	0.0003 (4)
Cl1	0.02788 (17)	0.02064 (16)	0.03483 (19)	−0.00701 (12)	−0.00165 (13)	0.00283 (13)
O3	0.0325 (5)	0.0191 (5)	0.0334 (6)	−0.0029 (4)	0.0071 (4)	−0.0023 (4)
O4	0.0290 (5)	0.0232 (5)	0.0250 (5)	−0.0049 (4)	0.0060 (4)	−0.0053 (4)
C15	0.0219 (6)	0.0215 (6)	0.0221 (6)	−0.0008 (5)	−0.0026 (5)	−0.0030 (5)
C16	0.0186 (5)	0.0205 (6)	0.0224 (6)	−0.0020 (5)	−0.0038 (5)	−0.0037 (5)
C17	0.0223 (6)	0.0171 (6)	0.0252 (7)	−0.0015 (5)	−0.0034 (5)	−0.0043 (5)
C18	0.0204 (6)	0.0213 (6)	0.0237 (6)	−0.0029 (5)	−0.0012 (5)	−0.0045 (5)
C19	0.0186 (5)	0.0195 (6)	0.0268 (7)	−0.0047 (5)	−0.0052 (5)	−0.0005 (5)
C20	0.0220 (6)	0.0174 (6)	0.0337 (7)	−0.0016 (5)	−0.0057 (5)	−0.0065 (5)
C21	0.0202 (6)	0.0228 (6)	0.0265 (7)	−0.0012 (5)	−0.0026 (5)	−0.0078 (5)
Cl2	0.03914 (19)	0.02243 (17)	0.02819 (18)	−0.01267 (13)	0.00685 (14)	−0.00548 (13)
O5	0.0297 (5)	0.0185 (5)	0.0339 (6)	−0.0007 (4)	0.0079 (4)	−0.0010 (4)
O6	0.0214 (4)	0.0210 (5)	0.0258 (5)	−0.0010 (4)	0.0039 (4)	−0.0023 (4)
C22	0.0199 (6)	0.0198 (6)	0.0206 (6)	−0.0004 (5)	−0.0027 (5)	−0.0027 (5)
C23	0.0199 (6)	0.0196 (6)	0.0184 (6)	−0.0018 (5)	−0.0025 (5)	−0.0037 (5)
C24	0.0218 (6)	0.0180 (6)	0.0247 (6)	0.0009 (5)	−0.0026 (5)	−0.0030 (5)
C25	0.0205 (6)	0.0233 (6)	0.0237 (6)	−0.0019 (5)	−0.0009 (5)	−0.0053 (5)
C26	0.0272 (6)	0.0207 (6)	0.0196 (6)	−0.0073 (5)	−0.0007 (5)	−0.0046 (5)
C27	0.0298 (7)	0.0167 (6)	0.0238 (6)	−0.0007 (5)	−0.0029 (5)	−0.0039 (5)
C28	0.0214 (6)	0.0200 (6)	0.0216 (6)	0.0010 (5)	−0.0022 (5)	−0.0059 (5)

### Geometric parameters (Å, º)

**Table:**

O1—C7	1.2307 (14)	C13—H13A	0.9900
N1—C1	1.3386 (17)	C13—H13B	0.9900
N1—C5	1.3445 (17)	C14—C14^ii^	1.539 (2)
N2—C7	1.3292 (15)	Cl1—C19	1.7402 (13)
N2—C6	1.4612 (16)	O3—C15	1.2172 (17)
N2—H2N	0.876 (9)	O4—C15	1.3196 (16)
C1—C2	1.3838 (19)	O4—H4O	0.847 (9)
C1—H1	0.9500	C15—C16	1.4958 (18)
C2—C3	1.3935 (17)	C16—C17	1.3938 (19)
C2—H2	0.9500	C16—C21	1.3957 (18)
C3—C4	1.3915 (18)	C17—C18	1.3861 (19)
C3—C6	1.5111 (17)	C17—H17	0.9500
C4—C5	1.3879 (19)	C18—C19	1.3886 (19)
C4—H4	0.9500	C18—H18	0.9500
C5—H5	0.9500	C19—C20	1.389 (2)
C6—H6A	0.9900	C20—C21	1.388 (2)
C6—H6B	0.9900	C20—H20	0.9500
C7—C7^i^	1.537 (2)	C21—H21	0.9500
O2—C14	1.2325 (14)	Cl2—C26	1.7418 (13)
N3—C12	1.3385 (17)	O5—C22	1.2173 (17)
N3—C8	1.3408 (17)	O6—C22	1.3181 (16)
N4—C14	1.3269 (15)	O6—H6O	0.846 (9)
N4—C13	1.4471 (16)	C22—C23	1.4945 (18)
N4—H4N	0.865 (9)	C23—C28	1.3923 (18)
C8—C9	1.3830 (19)	C23—C24	1.3977 (17)
C8—H8	0.9500	C24—C25	1.3850 (19)
C9—C10	1.3911 (17)	C24—H24	0.9500
C9—H9	0.9500	C25—C26	1.3871 (19)
C10—C11	1.3906 (18)	C25—H25	0.9500
C10—C13	1.5118 (17)	C26—C27	1.3862 (19)
C11—C12	1.3886 (19)	C27—C28	1.3869 (19)
C11—H11	0.9500	C27—H27	0.9500
C12—H12	0.9500	C28—H28	0.9500
			
C1—N1—C5	117.89 (11)	N4—C13—H13B	108.7
C7—N2—C6	121.67 (10)	C10—C13—H13B	108.7
C7—N2—H2N	119.5 (11)	H13A—C13—H13B	107.6
C6—N2—H2N	118.6 (11)	O2—C14—N4	125.67 (11)
N1—C1—C2	123.12 (12)	O2—C14—C14^ii^	121.76 (13)
N1—C1—H1	118.4	N4—C14—C14^ii^	112.57 (12)
C2—C1—H1	118.4	C15—O4—H4O	106.4 (14)
C1—C2—C3	119.15 (12)	O3—C15—O4	124.16 (12)
C1—C2—H2	120.4	O3—C15—C16	122.31 (12)
C3—C2—H2	120.4	O4—C15—C16	113.49 (11)
C4—C3—C2	117.87 (12)	C17—C16—C21	119.62 (12)
C4—C3—C6	121.98 (11)	C17—C16—C15	118.56 (12)
C2—C3—C6	120.14 (11)	C21—C16—C15	121.73 (12)
C5—C4—C3	119.37 (11)	C18—C17—C16	120.60 (12)
C5—C4—H4	120.3	C18—C17—H17	119.7
C3—C4—H4	120.3	C16—C17—H17	119.7
N1—C5—C4	122.59 (12)	C17—C18—C19	118.62 (12)
N1—C5—H5	118.7	C17—C18—H18	120.7
C4—C5—H5	118.7	C19—C18—H18	120.7
N2—C6—C3	112.69 (10)	C18—C19—C20	122.06 (12)
N2—C6—H6A	109.1	C18—C19—Cl1	118.50 (10)
C3—C6—H6A	109.1	C20—C19—Cl1	119.44 (10)
N2—C6—H6B	109.1	C21—C20—C19	118.52 (12)
C3—C6—H6B	109.1	C21—C20—H20	120.7
H6A—C6—H6B	107.8	C19—C20—H20	120.7
O1—C7—N2	125.33 (11)	C20—C21—C16	120.55 (12)
O1—C7—C7^i^	121.12 (13)	C20—C21—H21	119.7
N2—C7—C7^i^	113.55 (12)	C16—C21—H21	119.7
C12—N3—C8	117.64 (11)	C22—O6—H6O	108.1 (13)
C14—N4—C13	123.73 (10)	O5—C22—O6	124.16 (12)
C14—N4—H4N	117.5 (11)	O5—C22—C23	122.35 (12)
C13—N4—H4N	118.7 (11)	O6—C22—C23	113.49 (11)
N3—C8—C9	123.14 (12)	C28—C23—C24	119.54 (12)
N3—C8—H8	118.4	C28—C23—C22	121.62 (11)
C9—C8—H8	118.4	C24—C23—C22	118.84 (11)
C8—C9—C10	119.28 (12)	C25—C24—C23	120.48 (12)
C8—C9—H9	120.4	C25—C24—H24	119.8
C10—C9—H9	120.4	C23—C24—H24	119.8
C11—C10—C9	117.71 (12)	C24—C25—C26	118.68 (12)
C11—C10—C13	123.06 (11)	C24—C25—H25	120.7
C9—C10—C13	119.23 (11)	C26—C25—H25	120.7
C12—C11—C10	119.35 (12)	C27—C26—C25	122.06 (12)
C12—C11—H11	120.3	C27—C26—Cl2	118.96 (10)
C10—C11—H11	120.3	C25—C26—Cl2	118.97 (10)
N3—C12—C11	122.87 (12)	C26—C27—C28	118.61 (12)
N3—C12—H12	118.6	C26—C27—H27	120.7
C11—C12—H12	118.6	C28—C27—H27	120.7
N4—C13—C10	114.16 (10)	C27—C28—C23	120.62 (12)
N4—C13—H13A	108.7	C27—C28—H28	119.7
C10—C13—H13A	108.7	C23—C28—H28	119.7
			
C5—N1—C1—C2	0.7 (2)	O4—C15—C16—C17	172.82 (12)
N1—C1—C2—C3	−0.1 (2)	O3—C15—C16—C21	178.55 (13)
C1—C2—C3—C4	−0.79 (18)	O4—C15—C16—C21	−3.73 (18)
C1—C2—C3—C6	179.63 (11)	C21—C16—C17—C18	1.81 (19)
C2—C3—C4—C5	1.09 (18)	C15—C16—C17—C18	−174.81 (12)
C6—C3—C4—C5	−179.34 (12)	C16—C17—C18—C19	−0.24 (19)
C1—N1—C5—C4	−0.3 (2)	C17—C18—C19—C20	−1.41 (19)
C3—C4—C5—N1	−0.5 (2)	C17—C18—C19—Cl1	178.20 (10)
C7—N2—C6—C3	−101.17 (13)	C18—C19—C20—C21	1.4 (2)
C4—C3—C6—N2	76.87 (14)	Cl1—C19—C20—C21	−178.18 (10)
C2—C3—C6—N2	−103.57 (13)	C19—C20—C21—C16	0.20 (19)
C6—N2—C7—O1	2.55 (18)	C17—C16—C21—C20	−1.79 (19)
C6—N2—C7—C7^i^	−176.74 (11)	C15—C16—C21—C20	174.72 (12)
C12—N3—C8—C9	0.65 (19)	O5—C22—C23—C28	−163.13 (13)
N3—C8—C9—C10	0.33 (19)	O6—C22—C23—C28	17.41 (17)
C8—C9—C10—C11	−0.96 (18)	O5—C22—C23—C24	16.56 (19)
C8—C9—C10—C13	179.04 (11)	O6—C22—C23—C24	−162.89 (12)
C9—C10—C11—C12	0.65 (18)	C28—C23—C24—C25	−0.43 (19)
C13—C10—C11—C12	−179.36 (11)	C22—C23—C24—C25	179.87 (12)
C8—N3—C12—C11	−0.99 (19)	C23—C24—C25—C26	1.1 (2)
C10—C11—C12—N3	0.3 (2)	C24—C25—C26—C27	−0.9 (2)
C14—N4—C13—C10	−98.77 (13)	C24—C25—C26—Cl2	178.27 (10)
C11—C10—C13—N4	26.33 (16)	C25—C26—C27—C28	0.1 (2)
C9—C10—C13—N4	−153.67 (11)	Cl2—C26—C27—C28	−179.13 (10)
C13—N4—C14—O2	2.03 (19)	C26—C27—C28—C23	0.6 (2)
C13—N4—C14—C14^ii^	−177.41 (12)	C24—C23—C28—C27	−0.46 (19)
O3—C15—C16—C17	−4.90 (19)	C22—C23—C28—C27	179.24 (12)

Symmetry codes: (i) −*x*+1, −*y*+2, −*z*; (ii) −*x*+2, −*y*+2, −*z*.

### Hydrogen-bond geometry (Å, º)

**Table:**

*D*—H···*A*	*D*—H	H···*A*	*D*···*A*	*D*—H···*A*
N2—H2*N*···O1^i^	0.88 (1)	2.35 (2)	2.7088 (14)	105 (1)
N4—H4*N*···O2^ii^	0.87 (1)	2.30 (2)	2.7028 (14)	108 (1)
O4—H4*O*···N1	0.85 (2)	1.82 (2)	2.6559 (15)	171 (2)
O6—H6*O*···N3	0.85 (2)	1.80 (2)	2.6419 (15)	172 (2)
C8—H8···O5	0.95	2.53	3.1708 (17)	125
N2—H2*N*···O2^iii^	0.88 (1)	2.11 (1)	2.8800 (13)	146 (1)
N4—H4*N*···O1	0.87 (1)	2.07 (1)	2.7959 (13)	141 (1)
C2—H2···O3^iv^	0.95	2.53	3.3597 (17)	146
C6—H6*A*···O3^iv^	0.99	2.59	3.5262 (17)	157
C9—H9···O5^v^	0.95	2.59	3.3701 (17)	140
C13—H13*A*···O5^v^	0.99	2.48	3.4006 (16)	155
C27—H27···O1^vi^	0.95	2.53	3.4505 (16)	163

Symmetry codes: (i) −*x*+1, −*y*+2, −*z*; (ii) −*x*+2, −*y*+2, −*z*; (iii) *x*−1, *y*, *z*; (iv) −*x*, −*y*+2, −*z*+1; (v) −*x*+1, −*y*+2, −*z*+1; (vi) −*x*+1, −*y*+1, −*z*+1.
